# Sharing health data for research purposes: results of a population survey in Germany

**DOI:** 10.1186/s12913-025-12706-9

**Published:** 2025-05-14

**Authors:** Caroline Dotter, Sonja Haug, Rainer Schnell, Georgios Raptis, Karsten Weber

**Affiliations:** 1 OTH Regensburg, Regensburg, Germany; 2 Universität Duisburg-Essen, Duisburg, Germany

**Keywords:** Data sharing, Health informatics, Health data utilization, Research data management

## Abstract

**Background:**

Increased use of health data has the potential to improve both health care and health policies. Several recent policy initiatives at the European and German legislative levels aim to increase the primary and secondary use of health data. However, little is known about general population views on health data access for research. Most studies are based on subsets defined by specific illnesses.

**Methods:**

We commissioned a national computer-assisted dual-frame telephone survey (landline and mobile). Logit estimation models were used to identify predictors of willingness to provide access to health data to different organizations (universities in Germany, universities worldwide, German government organizations, pharmaceutical companies).

**Results:**

A high willingness to share health data for research purposes is observed, depending on the specific data recipient. The willingness is highest for research at universities in Germany and German governmental organizations, and lowest regarding research by pharmaceutical companies. The main drivers for sharing health data are the level of trust in public institutions, the respondents’ assessment of the seriousness and likelihood of data misuse, and the level of digital literacy. Age, gender, and level of education have small effects and do not determine the willingness to share health data for all organizations.

**Conclusion:**

We present evidence from a random sample of the German population. The results indicate widespread support among the population for providing access to health data for research purposes. Similar to findings in other countries, the willingness depends strongly on the recipient of the data. This paper evaluates the impact of various determinants – identified in previous qualitative and quantitative research – on the willingness of the German population to share health data. While previous studies have found that patients are generally more willing to share health data, we found that the presence of a medical precondition does not translate into respondents’ unequivocal support for health data sharing. We identify privacy concerns, general trust, and digital literacy as key factors influencing the willingness to share health data. Therefore, policymakers and stakeholders need to ensure and communicate the necessary privacy protection measures to increase the willingness of the German population to share health data.

## Background

### Motivation

Increased primary and secondary use of health data allows for better policymaking, more innovation in health care and, most importantly, improved quality of health care [1, 2]. The need to tap into this wealth of data has been recognized by policymakers, and legislation has recently been introduced at both European and national levels. The German government introduced a Health Data Utilization Act in the spring of 2024, which enables expanded use of health data and establishes a research data infrastructure by defining legal rules for the broader use of primary and secondary health data.

At the European level, the Commission’s proposal for a European Health Data Space (EHDS)^1^ as a platform for sharing and transferring health data across EU member states was adopted by the European Parliament and Council in spring 2024. This legislation sets rules for secure access to and processing of health data, establishes a health data infrastructure, and is intended to enable expanded primary and secondary use of health data across the EU.

Attitudes toward data privacy are not homogeneous across the EU, and the status of (health data) digitization also varies. In Germany, in particular, privacy is taken very seriously by data protection officials and the public. This is reflected, for example, in data protection legislation and several court rulings that complement the EU-wide GDPR. The European Union’s General Data Protection Regulation (GDPR) is also interpreted more strictly in Germany, for example regarding the linkage of multiple databases containing sensitive personal data ([3, 4], p.33). As a result, privacy concerns have historically limited the secondary use of data for research purposes in Germany [4]. The EHDS regulation will provide greater clarity on the interpretation of the GDPR and harmonize the conditions and permissions for primary and secondary use of health data across the European Union. This enables cross-border sharing and analysis of health data for research purposes within the boundaries of the GDPR. Given these ambitious (national and European) policy plans, knowing the attitudes of the population towards the use of health data is relevant to overcome the potential reluctance of the German population to use this data for research.

 To collect the data for the EHDS, electronic patient files are introduced in Germany. The adoption of mandatory electronic patient record (ePA) for all German citizens who are members of a statutory health insurance (which is a major part of the German adult population) will afford huge financial resources. Although the ePA must be provided by the insurance companies the insured can opt-out. Since high numbers of opt-outs could jeopardize these policy plans, potential reasons for opting out must be identified so that adequate measures can be taken to remedy them. Furthermore, introducing the ePA is expensive and must ultimately be covered by the insured. A failure due to widespread non-acceptance of the ePA would mean a fatal misallocation of resources.

The study presented here provides information concerning attitudes, preferences, and knowledge of the population. The results should inform stakeholders concerned with the implementation of the ePA. We studied the willingness and predictors to share health data in a random sample of the German population.

Previous results are mostly based on qualitative studies or used convenience samples of patients, as shown below. Empirical studies are necessary to understand public opinion on health data sharing and to overcome potential hurdles. Given the above-mentioned ambitious (national and European) policy plans, it is relevant to know the attitudes of the population towards the use of health data to possibly overcome the assumed reluctance of the German population in particular to use health data. Effective data governance policies need to appreciate and incorporate public perspectives. Therefore, we aim to study the willingness and predictors to share health data in a random sample of the German population.

### Previous research

The existing evidence on attitudes towards (sharing) health data is often qualitative and focused on countries with an existing health data infrastructure (e.g., UK, Nordic countries). Systematic and narrative literature reviews show strong evidence that patients and the public support the sharing of personal health data [5–9]. However, support appears to be conditional on several factors including type of data user, type of data, transparency and individual control [5, 6]. Patients are often more willing than the general public to share health data, suggesting that expectations of (personal) benefit may influence the willingness to share health data. In addition, this group may perceive more benefits than the general public [8].

However, the willingness to share personal health data is often found to depend on sufficient anonymization, control over the data through different consent models such as opt-in and opt-out, trust in the organizations storing and processing the data, and the intentions of the data users [5]. Data sharing is accepted when a public and/or personal benefit for the individual sharing the data is identified. More public or societal benefits appear to balance or even outweigh privacy concerns [5].

The available qualitative evidence suggests that the involvement of the private sector (e.g., pharmaceutical companies) is being viewed in a more nuanced manner. The goal of private companies is considered to be profit rather than public welfare [10]. The purpose and potential benefit of sharing health data is crucial for acceptance of data sharing, as a focus group study on people in England, Iceland and Sweden found [11]. Thus, privacy is given preference in situations where private companies are involved, as societal benefits are less obvious. Trust in a particular organization plays an even greater role when the private sector is involved [12, 13]. The level of trust individuals have in potential data users (e.g., research organizations such as universities as well as pharmaceutical companies, etc.) and respective oversight bodies affect their support for the employment of health data in research. Trust is generally higher for individuals and organizations people are familiar with [5].

These results are supported by evidence of discrete choice experiments: Evidence for 12 European countries (Austria, Denmark, France, Germany, Iceland, Ireland, Italy, the Netherlands, Norway, Spain, Sweden, and the United Kingdom) shows people want information and want to exert control over data use. Private enterprises are considered least acceptable for collecting and using health data [14]. Research conducted in Australia has indicated a general disinclination toward the involvement of private companies, as well as a need to provide explicit information regarding the intended purpose of data collection, the limitations imposed on its usage, and the restrictions on access [15].

Depending on the research method (qualitative vs. quantitative), the population, and the setting of the respective surveys, findings differ on what factors influence individuals’ decisions (not) to share health data. Systematic comparisons are limited as most quantitative studies focus on specific populations (e.g., patients with a specific condition) and assess very specific data sharing scenarios (e.g., data sharing for genomic research or in a public health context). In addition, the covariates of interest vary across studies. Consequently, the ensuing comparison across studies must be regarded with a degree of caution.

One variable discussed in most studies is the age of potential data donors. The direction of the effect is, however, unclear [6]. Evidence also exists on the non-linearity of the effect: In several studies, both younger (< 25/< 35) and older (> 55/> 65) people are in favor of sharing health data [9]. Retirees have also been found to support health data sharing [16]. Older people may see more personal benefits from potential advances in medicine due to the availability of health data. Concurrently, this demographic may be less cognizant of the potential for data leakage. In addition, they do not have to worry about data breaches affecting their job prospects [16].

The impact of other socioeconomic factors on respondents’ willingness to share personal health information varies. Depending on the study, people with less education are either more or less likely to support data sharing [6]: A quantitative study of the Irish general population found that higher levels of education were associated with a lower likelihood of allowing researchers access to health data without obtaining consent [16]. Similar results were found in a UK study (convenience sample of potential patients), where respondents without academic qualifications were less concerned about data security [10]. On the other hand, another UK study, also using a convenience sample of patients, found that respondents with lower levels of education were more likely to expect to be asked for explicit consent [17].

Most studies focus on populations in countries with more extensive access to health data than Germany (e.g., UK, Ireland, USA, etc.). One study compared the willingness of German speakers to donate medical and DNA information with English speakers and found that German participants were less willing to share their data [18]. Conversely, a Europe-wide vignette study found that health privacy concerns in Germany were in the medium and low range compared to other European countries and lower than in the United Kingdom and Ireland [19]. Additionally, a longitudinal online vignette study in Germany found that acceptance of sharing health data collected by wearables increased during the corona pandemic [20].^2^

However, it is unclear how strong health privacy concerns are in Germany considering the new national and proposed European legislation, and it is unclear how findings from other countries can be applied to the German situation. This paper analyzes the attitudes of the German population toward health data sharing and aims to identify determinants of willingness to share health data. It is hypothesized that attitudes toward data access vary according to the type of organization.

## Methods

### Survey

The questionnaire was designed based on a review of previous questionnaires on the following topics: attitudes toward electronic health records, acceptability of health data sharing, and consent preferences [21–23]. The items were supplemented by several sociodemographic questions including a single-item self-rated health measure derived from the German General Social Survey (ALLBUS) [24]. These are complemented by questions on trust in public institutions and political attitudes extracted from the World Values Survey [25].

The final^3^ questionnaire contains 46 questions and 69 items on several topics in the realm of digitization of health services, health data storage, and questions on attitudes and socio-demographics [26]. This paper focuses on willingness to share health data for research purposes as the dependent variable. The wording in the questionnaire (based on [23]) reads as follows:

Which organizations would you give access to health data if no inference can be made about you? (Response options: universities in Germany; universities around the world; government agencies involved in health research, for example, the Robert Koch Institute (RKI)^4^; pharmaceutical companies; none of these organizations; don’t know; not specified.)

### Sample

A nationwide computer-assisted dual-frame telephone survey (landline and mobile) was fielded between June 1, 2022 and June 27, 2022. The study is based on a simple random sample of the participants of the *institute for social science (infas)* multi-topic survey recruited via ADM telephone sample. The random sample of adult people living in Germany includes 1308 persons (43.6% female, 56.2% male, 0.2% (2) diverse, average age 61.6). In the following analysis, data is weighted^5^ to the known population profile.

### Analysis

Data analysis was performed with the *R Project for Statistical Computing* Version 4.2.1 using the packages survey, tidyverse, psych, stats, ggsurvey, and ggplot2. First, we describe the sample and univariate results of our dependent variable, the willingness to share health data for research purposes. Second, we explain how we capture potential determinants of the willingness to share health data. Finally, we present results of four logit models to explain the willingness to share health data with different recipients.

## Results

### Respondent characteristics

Between June 1 and 27, 2022 a total of 1,308 people aged 18 and above were surveyed by telephone. The weighted sample consists of 50,8% female, 48,3% male, 0,8% diverse respondents. Respondents are on average 51, 67 years (std.: 18,94) and 18% report having a university degree.

### Willingness to share health data

Respondents’ willingness to share health data is high but depends on the recipient. While the vast majority are in favor of sharing anonymized health data for research at German universities (91.2%), there is less support for sharing health data for research at universities worldwide (50.5%). There is a high willingness to share health data with government agencies involved in health research (e.g., RKI) (86.3%). However, respondents are less willing to share health data with pharmaceutical companies (30.3%) (see Table 1). Similar to qualitative findings for other countries, respondents in Germany seem to prefer sharing health data with organizations in their country rather than with organizations in other countries. Sharing health data is even less supported in case of pharmaceutical companies.

**Table 1:** Willingness to share health data with organizations^a^

	n	Percent of cases
Organizations		
Universities in Germany	1185	91.2
Universities worldwide	657	50.5
Government agencies in health research	1122	86.3
Pharmaceutical companies	396	30.5
No such organization	39	3.0

^a^Question: Which organizations would you give access to health data if no inference can be made about you?

### Explanatory variables

Based on the literature discussed above, we assume that the willingness to share health data is determined by gender, age, education, health status, likelihood and perceived harmfulness of data leakage, variables reflecting respondents’ level of digital literacy, and questions about trust in public institutions.

#### Education

The survey asked for the respondent’s level of education: Whether they have a tertiary degree, and what form of highest schooling they have completed. Based on the answers to these two questions, we generated a new internationally comparable education variable that reflects the levels of the International Standard Classification of Education (ISCED) 2011. Previous findings on the effect of education on willingness to share health data have been ambiguous [9].

#### Health

Respondents were asked to rate their health on a scale of 1 (very good) to 5 (poor). Findings in the literature suggest that people in poorer health and with chronic conditions are more likely to share health data because they might expect potential (personal) benefits [8]. While self-rated health is an imperfect indicator of respondents’ true health status^6^, we consider the variable as a proxy, as the respondent’s self-perception as a potential patient might influence their willingness to share data. Most respondents consider themselves to be in good health (44%), only 5% consider themselves to be in very poor health, and no respondent considers themselves to be in very good health.

#### Digital literacy

In the survey, two questions relate to respondents’ use of digital devices: Respondents were asked which digital devices they use (multiple choices or none) and which devices they use to access the Internet (multiple choices or none). A growing body of literature provides evidence that the mode of access is a strong indicator of the type of use: Mobile (smartphone/tablet) access to the Internet is mostly used for entertainment purposes. Yet, to fully exploit the potential of digital services and achieve high levels of digital literacy, multimodal access to the internet is required [29–33]. Accordingly, we generated a new indicator variable that reflects the respondent’s level of digital literacy: 0 = no access to the Internet, 1 = access to the Internet only via smartphone and/or tablet, 2 = access to the Internet via PC or laptop. Additionally, we included the variable “experience with videocalls”. This is a binary variable capturing respondents reported experience with videocalls.

#### Trust

The level of trust individuals have in data users and their respective oversight bodies influences their support for research use of health data [5]. The questionnaire asks about trust in ten different organizations on a ten-point scale [25]. Some of these organizations are potential data users, others are potential oversight bodies. Cronbach’s alpha for these ten items is very high (0.92). There is little agreement on measures of general institutional trust in the literature.^7^ Here, we decided to focus on trust in public administration and universities, as these are the most likely data users and oversight institutions. In a previous study, trust in universities was associated with a general willingness to share health data [36].

#### Fears of data leakage

Since increased trust supports willingness to share health data for research, we expect that increased fears of data leakage should negatively affect support for research use of health data. Three questions related to respondents’ fears about the possibility of data leakage: One question asks how often the respondent worries about malicious software generally; the other two questions are specific to health data, asking how likely and how serious respondents think misuse of health data is. We include all three in our initial set of explanatory variables.

### Multivariate results

The variables discussed above should determine the respondent’s attitude toward sharing data with different organizations.

Our analysis for the example of Germany confirms qualitative and quantitative findings regarding other countries with increased access to health data: Respondents’ willingness to share health data depends on the recipient. People are more willing to share health data with organizations they know and trust (see Table 1).

Table 2 shows the logistic regression results for the willingness to provide access to health data to four different organizations. Covariate effects are reported as odds ratios with confidence intervals. The covariate effects^8^ explaining individuals’ willingness to share health data vary in magnitude and direction for the different data recipients. The models for the different organizations also differ in their model fit: The model explaining willingness to provide access to health data to universities in Germany has the best model fit.

Age only has a significant effect on the willingness to provide access to health data to universities worldwide. Gender only affects the willingness to provide access to pharmaceutical companies: Women are more likely to give access to health data to pharmaceutical companies than respondents who identify as male.

The education of the respondents has a significant negative effect on the willingness to share health data with universities in Germany and pharmaceutical companies. The effect of subjective health on respondents’ willingness to provide access to health data differs across organizations. While a worse health status decreases the willingness to give access to pharmaceutical companies, it increases the willingness to give access to universities worldwide and government agencies (such as the RKI).

The two variables that capture respondents’ digital literacy have opposite effects. Experience with video calls has a strong positive effect on willingness to share with all organizations except pharmaceutical companies. The variable “level of digitization” is an indicator variable reflecting the number of different devices used by respondents. The number of devices used has a strong negative effect on the willingness to provide access to health data to government agencies, but not to other organizations.

**Table 2:** Logistic regression results (reported odds-ratios and 95%confidence intervals)

	Universities in Germany	Universities worldwide	Governmental agencies (RKI)	Pharmaceutical companies
(Intercept)	0.12	0.35	10.82	1.51
	[0.01, 1.32]	[0.09, 1.35]	[0.94, 142.89]	[0.37, 6.15]
Age	1.01	0.98***	1	0.99
	[1, 1.03]	[0.97, 0.99]	[0.99, 1.02]	[0.99, 1]
Gender [base = male]	0.79	1.26	1.17	1.34*
	[0.46, 1.33]	[0.95, 1.67]	[0.75, 1.84]	[1, 1.78]
Level of education	0.67*	0.95	0.99	0.79*
	[0.47, 0.99]	[0.79, 1.14]	[0.73, 1.34]	[0.65, 0.96]
Subjective health	0.87	1.24*	1.38*	0.75**
	[0.65, 1.16]	[1.04, 1.49]	[1.03, 1.87]	[0.62, 0.91]
exp_videocall [base = no]	4.02***	2.14***	3.85***	0.74
	[2.21, 7.49]	[1.53, 3.01]	[2.33, 6.47]	[0.52, 1.06]
Level of digitization	1.29	0.9	0.11***	1.28
	[0.62, 2.64]	[0.57, 1.41]	[0.04, 0.27]	[0.8, 2.07]
Data misuse	0.92*	0.93**	0.92*	0.94**
	[0.85, 1]	[0.89, 0.98]	[0.86, 0.98]	[0.9, 0.98]
Fears of malware	1.78***	1.06	0.95	0.85*
	[1.37, 2.36]	[0.92, 1.22]	[0.78, 1.16]	[0.73, 0.98]
Trust	1.93***	1.34***	1.72***	1.15**
	[1.62, 2.33]	[1.22, 1.47]	[1.49, 2.01]	[1.04, 1.26]
	1000	1000	1000	1000
AIC	398.4	1093.2	504.6	1047.2
BIC	447.5	1142.3	553.6	1096.2
Log.Lik.	− 189.19	− 536.59	− 242.28	− 513.58
RMSE	0.26	0.48	0.32	0.43

* *p* < 0.05, ** *p* < 0.01, *** *p* < 0.001

The respondents’ fear of data leakage is reflected by two variables, one assessing the frequency of the respondents’ fear of malware (scale 1–5, 5 = very often) and a multiplicative index generated from two questions asking for the individual’s assessment of the probability and harmfulness of data leakage for personal health data (“data misuse”).

Fear of malware affects the willingness to share health data differently for different organizations. Respondents who worry more often about malware are more willing to give universities in Germany access to their health data than respondents who worry less often. In contrast, respondents who worry more often about malware are less willing to give pharmaceutical companies access.

Finally, the indicator variable “trust” has a strong positive and significant effect on the willingness to give access to any organization. The variable reflects general trust in public organizations, as it is the average trust level for 10 organizations (on a 10-point scale). The effect is strongest for the willingness to give access to universities in Germany and government organizations.

## Discussion

The study aims to observe the population’s willingness to share health data in Germany. We observe that respondents are more likely to share their health data with German organizations, universities, as well as government organizations.

We can assume that these organizations are better known to the population. The organization types “universities worldwide” and “pharmaceutical companies” are broader and more ambiguous. Respondents may assume that the benefits from research of these types of organizations have no positive impact on them. This is supported by evidence from a systematic review that found data sharing preferences strongly depend on the data user: its motivation, trust in the data user, and whether it is a for-profit organization [6]. It is consistent with cross-country evidence utilizing a discrete choice experiment: Data sharing is most acceptable for all types of respondents if a national authority or academic research project is the data user and least acceptable for private enterprises to collect or use their digital health data [14].

A cross-country survey (Denmark, Sweden, The Netherlands, and the United Kingdom) of people with diabetes also specifically asked for the organization type “global universities” and found high acceptance for data sharing with this type of organization. They observed a consistently high willingness to share data with European universities, global universities, and government-funded organizations (89.8%, 86.8%, 87.2%), and a lower rate (56.4%) for commercial research companies [23].

Socio-demographic determinants influence individuals’ willingness to share health data. The older respondents are, the less willing they are to provide access to universities worldwide. For all other organizations, age has no significant effect on willingness to provide access to health data. As age interacts with health and level of digitization [37], our multivariate analysis may account for this effect. Women are more likely to give access to health data to pharmaceutical companies than respondents who identify as male. For all other organizations, gender has no effect on willingness to share health data. Respondents with higher levels of education are less willing to give access to their health data to universities in Germany and pharmaceutical companies. For the other two organizations, education level has no effect.

Poor subjective health generally increases the willingness to share health data, except for pharmaceutical companies. Several previous studies have focused on patient populations. These studies generally found greater willingness among patients than among the general public [8]. Our findings are not as straightforward, as the effect of subjective health varies across organizations: Poor subjective health increases willingness to provide access to universities worldwide and to government agencies but decreases willingness to provide access to health data to pharmaceutical companies. This surprising result may be explained by a nuanced view of private sector involvement in data sharing since their goal is considered to be private profit [10].

Increased digital literacy, as measured by respondents’ experience with video calls, has a strong positive effect on willingness to provide access to all types of organizations except pharmaceutical companies. This is consistent with the qualitative evidence discussed above: Privacy is valued more highly concerning the private sector, as the goals of the private sector are considered to be not in the public interest [10, 12, 13].

We observe that if the misuse of personal health data is perceived to be more serious and likely, respondents are less willing to give access to their health data to any organization. This effect is negative and significant for all organizations. A high perception of the seriousness and probability of health data misuse has a strong negative effect on the willingness to share health data. This effect holds for all organizations. In contrast to this, respondents’ fear of malware affects the willingness to share health data only for universities in Germany and pharmaceutical companies. Fear of malware increases the willingness to give universities in Germany access to health data and reduces the willingness to give pharmaceutical companies access.

General trust in organizations increases the willingness to give access to any organization. Previous studies have asked about trust in specific data users and oversight bodies and found that trust positively influences willingness to share health data with them. Our findings support this, as we observe a strong and positive effect of trust in public institutions on willingness to share health data. People who trust public institutions more are more willing to share their health data with any type of organization. Thus, data sharing is only supported when trust in public institutions exists.

## Conclusion

We observe a strong willingness to share health data among the German population that depends on the recipient of the data: Universities in Germany and German government agencies receive high approval. These results are remarkable, since they are in contrast to the strong emphasis on privacy generally observed in Germany.

The willingness to share health data seems to be mainly driven by the level of trust in public institutions and the assessment of the seriousness and probability of data misuse. More trust in public institutions leads to a higher willingness to share health data, and a higher assessment of the seriousness and probability of data misuse reduces the willingness to share health data. Thus, the perceived probability of data misuse has a significant negative effect on the willingness to share health data. Trust and data security are critical issues in the public’s evaluation of data sharing practices.

Another important determinant is digital literacy, which is reflected in respondents’ experience with video calls. Having experience with video calls reduces the willingness to share health data with pharmaceutical companies but increases the willingness to share health data with all other organizations. Digital literacy is expected to result in the realistic assessment of data misuse. Therefore, increasing digital literacy might result in a reduction of unfounded fears of data misuse. Finally, implementing and communicating state-of-the-art privacy-enhancing technologies and technological and organizational measures for data protection might reduce privacy concerns.

## Data Availability

The dataset analysed during the current study is available from the corresponding author on reasonable request.

## Abbreviations

RKI: Robert Koch Institute
ISCED: International Standard Classification of Education

## Footnotes

1 Proposal for a regulation of the European Parliament and of the Council on the European Health Data Space; COM/2022/197 final.

2 Health data in this study however did not refer to medical data stored by doctors, but referred to health data collected by smartwatches etc.

3 Pretests of the questionnaire were performed by colleagues, in a university course and by the survey institute.

4 The Robert Koch Institute (RKI) is Germany’s federal institute for public health: www.rki.de.

5 Weighting was done in two steps: In the first step, default weights were calculated according to gender, age groups, education, and geographic location. In a second step, these weights were adjusted according to their probability of non-response using iterative proportional fitting, resulting in the final calibrated sample weights. The calibration step included age group, gender, household size, education, occupation and employment status, nationality, state, and municipality size class.

6 Self-rated health reflects personal perceptions of health that are constructed through health outcomes, psychological filters, and social or group aspects. To some extent, it is determined by the respondent’s personal outlook on life [27]. In addition, there is empirical evidence that people with chronic conditions adjust to being in poor health [28].

7 Some studies use single-item indicators, such as trust in parliament, while others use additive or multiplicative indices on a range of trust variables. Some argue there are different dimensions of trust, for instance trust in institutions that implement regulations or institutions that represent the public [34]. Furthermore, it must be noted that trust in the government and the parliament might actually reflect support for incumbent officeholders [35].

8 For ease of presentation, covariates are not shown at factor levels. Including factor levels slightly improves the model fit.
